# A Modular Coassembly Approach to All-In-One Multifunctional Nanoplatform for Synergistic Codelivery of Doxorubicin and Curcumin

**DOI:** 10.3390/nano8030167

**Published:** 2018-03-15

**Authors:** Muyang Yang, Lixia Yu, Ruiwei Guo, Anjie Dong, Cunguo Lin, Jianhua Zhang

**Affiliations:** 1 Department of Polymer Science and Technology, Key Laboratory of Systems Bioengineering (Ministry of Education), School of Chemical Engineering and Technology, Tianjin University, Tianjin 300072, China; yangmuyang@tju.edu.cn (M.Y.); lixiayu@tju.edu.cn (L.Y.); rwguo@tju.edu.cn (R.G.); ajdong@tju.edu.cn (A.D.); 2 Collaborative Innovation Center of Chemical Science and Engineering (Tianjin), Tianjin 300072, China; 3 State Key Laboratory for Marine Corrosion and Protection, Luoyang Ship Material Research Institute (LSMRI), Qingdao 266101, China; lincg@sunrui.net; 4 Tianjin Key Laboratory of Membrane Science and Desalination Technology, Tianjin University, Tianjin 300072, China

**Keywords:** modular coassemble, synergistic codelivery, polymeric prodrug, stimulisensitive release, biocompatibility

## Abstract

Synergistic combination therapy by integrating chemotherapeutics and chemosensitizers into nanoparticles has demonstrated great potential to reduce side effects, overcome multidrug resistance (MDR), and thus improve therapeutic efficacy. However, with regard to the nanocarriers for multidrug codelivery, it remains a strong challenge to maintain design simplicity, while incorporating the desirable multifunctionalities, such as coloaded high payloads, targeted delivery, hemodynamic stability, and also to ensure low drug leakage before reaching the tumor site, but simultaneously the corelease of drugs in the same cancer cell. Herein, we developed a facile modular coassembly approach to construct an all-in-one multifunctional multidrug delivery system for the synergistic codelivery of doxorubicin (DOX, chemotherapeutic agent) and curcumin (CUR, MDR modulator). The acid-cleavable PEGylated polymeric prodrug (DOX-*h*-PCEC), tumor cell-specific targeting peptide (CRGDK-PEG-PCL), and natural chemosensitizer (CUR) were ratiometrically assembled into in one single nanocarrier (CUR/DOX-*h*-PCEC@CRGDK NPs). The resulting CUR/DOX-*h*-PCEC@CRGDK NPs exhibited several desirable characteristics, such as efficient and ratiometric drug loading, high hemodynamic stability and low drug leakage, tumor intracellular acid-triggered cleavage, and subsequent intracellular simultaneous drug corelease, which are expected to maximize a synergistic effect of chemotherapy and chemosensitization. Collectively, the multifunctional nanocarrier is feasible for the creation of a robust nanoplatform for targeted multidrug codelivery and efficient MDR modulation.

## 1. Introduction

Cancer is a leading death cause worldwide, and thus an urgent need constantly exists to improve its therapeutic outcomes [1]. Currently, chemotherapy with the advantages of minimal invasion and convenient administration is still regarded to be one of the most appropriate strategies to treat cancer patients [2,3]. However, the chemotherapeutic efficacy, especially the single-agent chemotherapy, is still far from satisfactory. In addition of the complicated pathogenesis and diversified pathologies of cancers, the major reason for treatment failure can be ascribed to the intrinsic drawbacks of chemotherapeutics [3,4,5,6,7]. For example, most anti-cancer drugs often suffer from poor water solubility and low stability, causing low bioavailability and poor pharmacokinetics. In addition, chemotherapeutic agents are unable to discriminate between cancerous and normal cells, resulting in non-specific distribution, and thus severe systemic toxicity. Moreover, the development of multidrug resistance (MDR) that is associated with the overexpression of P-glycoproteins (P-gp) poses a tremendous challenge to effective cancer therapy [5,8]. Therefore, numerous efforts are still required to address these challenges and improve chemotherapeutic efficacy.

During the past decades, in view of the complexity of cancer and thus the limited clinical efficacy of monotherapy, the administration of two or more drugs with complementary anticancer mechanisms, i.e., combination chemotherapy, has become increasingly important to enhance anticancer efficacy [9,10,11,12,13]. In particular, the nanoparticulate combination therapy, i.e., codelivery of multiple drugs by nanoparticles (NPs), merged the beneficial features of both nanoformulations and combination therapy, which have demonstrated to be an effective strategy to treat cancer [12,14,15,16,17]. When compared with the conventional cocktail chemotherapy, the codelivery of multiple drugs in one NP system can realize the definitive delivery of a correct ratio of each drug and enable the controlled release in target site, prolong drug circulation half-life, accumulate at the tumors through enhanced permeation and retention (EPR) effect, and thus optimize the pharmacokinetics and biodistribution of drugs, achieving a more significant synergistic anticancer effect [14,15,16,17]. For example, the concurrent delivery of doxorubicin (DOX, one of the most active chemotherapeutics for cancer) and curcumin (CUR, a natural chemosensitizer with distinguishing abilities to inhibit P-gp overexpression and nuclear transcription factor NF-κB activation that are both closely linked to MDR) in one nanocarrier has been widely explored as an effective way to improve DOX treatment efficiency [18,19,20,21]. A variety of nanoparticulate delivery systems, such as liposomes or lipid NPs [21,22,23,24], polymeric micelles [25,26,27], prodrug NPs [28,29,30], as well as inorganic NPs [31,32,33], have been developed as codelivery vesicles for DOX/CUR. The codelivery of DOX/CUR with nanotechnology has achieved significant success in improving the therapeutic efficacy of DOX [18,19,20,21]. However, the nanoparticle-assisted combination therapies are still far from fulfilling their potential and problems still exist in many aspects [14,15,16,18,34].

Firstly, due to the different physicochemical profiles and distinct solubility characteristics, it remains a great challenge to coencapsulate high payloads of chemotherapeutic agents and chemosensitizers into a single NP to unify their pharmacokinetics and biodistribution, and also to ensure no drug leakage and no rapid clearance into reticuloendothelial organs during in vivo transportation [14,15,18]. For example, the liposomes and polymeric micelles for multidrug codelivery often suffer from their low encapsulation capacity, instability, and premature drug leakage, meanwhile the applications of inorganic NPs frequently lead to their low biocompatibility and clearance of the immune system [3,4,35]. Another major challenge of the multidrug codelivery is the ability to improve the specificity to the disease site. Currently, most of the existing nanocarriers for combination therapy lack selectivity and targeting ability. Thus, their delivery into cancer tissues mostly relies on the EPR effect by the leaky tumor vasculatures varying among patients and their tumor types [36,37]. The active targeting, i.e., ligand-mediated targeting, involves using affinity ligands on the NPs surface for specific retention and uptake by tumor cells, which has been proven to be a promising complementary strategy for EPR in order to further improve the efficiency of cancer nanomedicines [36,38,39,40]. Apparently, the actively targeted NPs for combination drug delivery, without tedious steps for preparing to assure future scale-up, are in urgent need for enhanced therapeutic activity and reduced damage to healthy tissue. Finally, the nanocarriers should be able to effectively corelease the encapsulated chemotherapeutic drug and chemosensitizer into the same cancer cell at the same time after systemic drug administration, as the simultaneous intracellular corelease is the prerequisite for generation of synergistic responses [7,14,18]. Summarily, due to the different physicochemical and pharmacokinetic profiles, the synergistic codelivery of a chemotherapeutic agent and chemosensitizer will necessarily require further development of multifunctional nanocarriers with the capability of efficient encapsulation, targeted delivery, and intracellular controlled corelease.

**Scheme 1:**
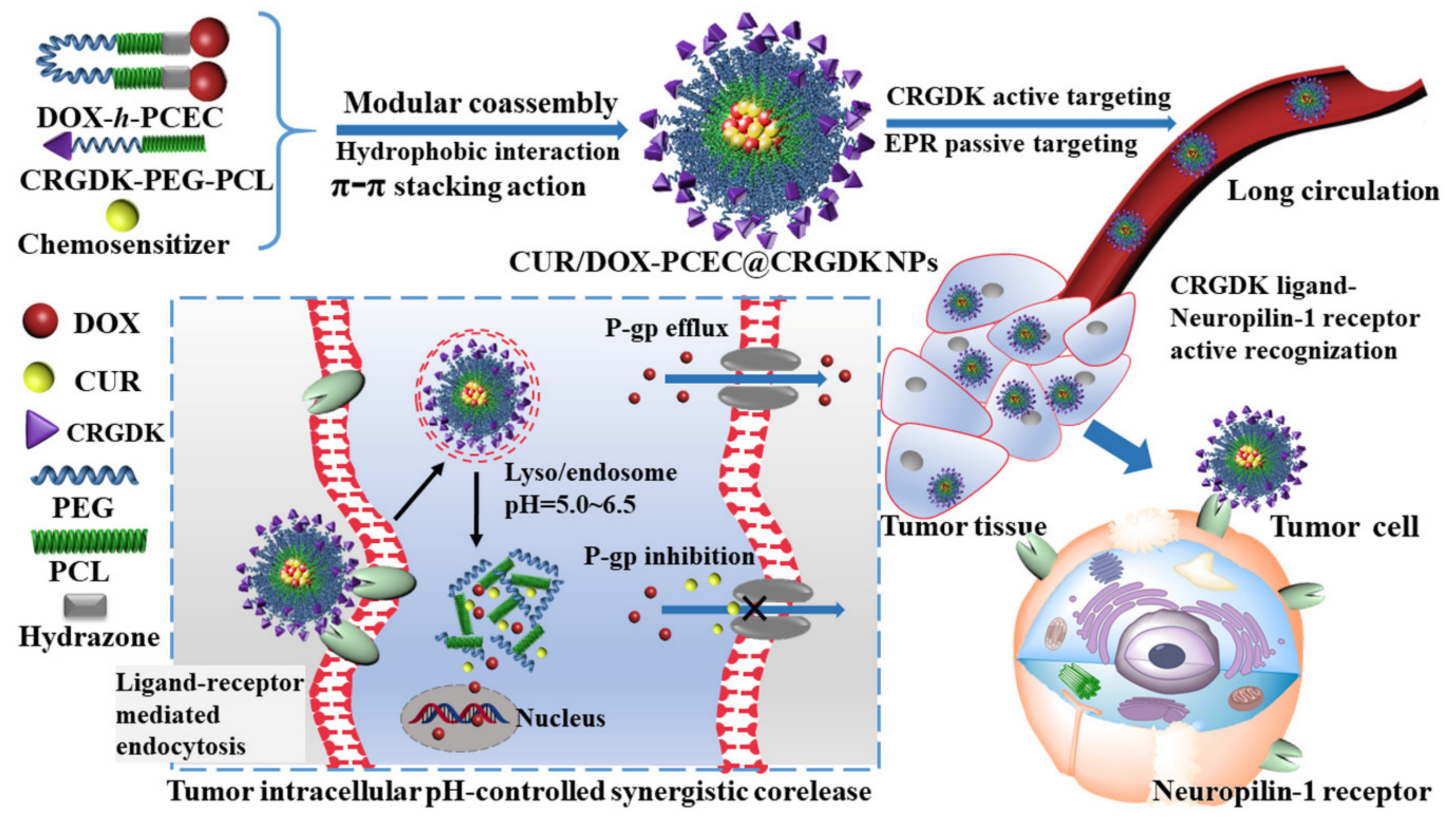
Schematic illustrations of the modular coassembly to construct an all-in-one multifunctional multidrug delivery system (curcumin (CUR)/ acid-cleavable PEGylated polymeric prodrug (DOX-*h*-PCEC) @Cys−Arg−Gly−Asp−Lys (CRGDK) nanoparticles (NPs)) with the capability of efficient encapsulation, targeted delivery, and intracellular controlled corelease.

In this study, a modular coassembly of acid-cleavable PEGylated polymeric prodrug, tumor cell-specific targeting peptide and natural chemosensitizer was designed and developed to construct multifunctional multidrug delivery system, as shown in Scheme 1. The chemotherapeutic agent DOX was conjugated to both ends of a biocompatible and biodegradable poly(ε-caprolactone)-*b*-poly(ethylene glycol)-*b*-poly(ε-caprolactone) (PCEC) via acid-cleavable hydrazone linkages, generating acid-cleavable PEGylated polymeric prodrug (DOX-*h*-PCEC) as drug vector model. To further introduce a tumor targeting capacity, cell-penetrating peptide (Cys−Arg−Gly−Asp−Lys, CRGDK) decorated poly(ethylene glycol)-*b*-poly(ε-caprolactone) (CRGDK-PEG-PCL, as active targeting model) was prepared via a maleimide-thiol reaction between CRGDK and maleimide-terminated PEG-PCL. CRGDK-mediated targeting can actively recognize the corresponding receptors (neuropilin-1 receptor, a transmembrane receptor glycoprotein overexpressed on the surface of a wide variety of tumor cells), which has been widely demonstrated to be able to increase the affinity for tumor cells and facilitate the drug-loaded NPs to efficiently enter the cells through ligand-receptor mediated endocytosis [41,42,43,44,45]. The DOX-*h*-PCEC and CRGDK-PEG-PCL are amphiphilic, and thus can coassemble in water, together with hydrophobic natural chemosensitizer (CUR, as MDR modulator model), forming a DOX/CUR coencapsulated, tumor-targeted, and intracellular acid-responsive nanoparticulate combination therapy system (CUR/DOX-*h*-PCEC@CRGDK NPs). With such unique structure, composition, and fabrication method, the CUR/DOX-*h*-PCEC@CRGDK NPs are expected to possess several desired functions: (1) high and tunable loading capacity derived from the PCL hydrophobic core and DOX hydrophobic phase, allowing for hydrophobic interaction and strong intermolecular π–π stacking action between DOX and CUR; (2) high stability and low leakage at physiological pH due to the combination of chemical conjugation and physical interactions; (3) cancer targeted delivery by the EPR effect and the active targeting of CRGDK; (4) stealth-shielding and long circulation from PEG shell; and, (5) intracellular simultaneous corelease of DOX and CUR through acid-triggered degradation of prodrug and subsequent disassembly of NPs to maximize a synergistic effect. Notably, the modular coassembly strategy to these all-in-one multifunctional NPs for DOX/CUR codelivery is cost-effective, as the complicated reactions and tedious synthesis processes for incorporating the desired multifunctionalities were minimized. Collectively, the design presented here provides a facile and robust nanoplatform for the targeted multidrug codelivery to maximize synergistic effects, holding great promises for the combinatory cancer therapy.

## 2. Materials and Methods

### 2.1. Materials

Poly (ethylene glycol) (PEG, *M*_n_ = 1500 Da), ε-caprolactone (CL, 99%) and tert-butyl carbazate (Boc-NHNH_2_, 99%) were purchased from Aladdin Industrial Co., Ltd. (Shanghai, China). Stannous octoate (Sn(Oct)_2_, 99%) was obtained from Sigma-Aldrich China (Shanghai, China). 4-Nitrophenyl chloroformate (NPC, 99%), trimethylamine (TEA, 99%), and trifluoroacetic acid (TFA, 99%) was purchased from J&K chemical Co. Ltd. (Beijing, China). Doxorubicin (DOX, 99%) and curcumin (CUR, 99%) were purchased from Dalian Meilun biotechnology Co., Ltd. (Dalian, China). Maleimide-poly(ethylene glycol)- hydroxy (Mal-PEG-OH) with the molecular weight of 2000 was purchased from JenKem Technology Co., Ltd. (Beijing, China). Cys–Arg–Gly–Asp–Lys (CRGDK, 99%) peptide was provided by GL Biochem. Ltd. (Shanghai, China). Dichloromethane (DCM, 99%), dimethyl sulfoxide (DMSO, 99%), tetrahydrofuran (THF, 99%), and other reagents were commercially available from Damao Chemical Co., Ltd. (Tianjin, China). *N*,*N*-dimethylformamide (DMF, 99%) for HPLC Analysis was purchased form Merck (Darmstadt, Germany).

The Neuropilin-1 overexpressed human umbilical vein endothelial cells (HUVEC) and Adriamycin resistant breast cancer cells (MCF-7/ADR) were obtained from Procell life science and technology Co., Ltd. (Wuhan, China).

### 2.2. Synthesis and Characterization of DOX-h-PCEC and CRGDK-PEG-PCL

The hydrazone-linked DOX polymeric prodrug DOX-hydrazone-poly(ε-caprolactone)-*b*-poly(ethyleneglycol)-*b*-poly(ε-caprolactone)-hydrazone-DOX (DOX-*h*-PCEC) was prepared in three steps, according to a modified procedure [46], including (I) ring-opening polymerization of CL with HO-PEG-OH to prepare PCEC based on our group’s previous work [47]; (II) introduction of the hydrazide group to both ends of PCEC; and, (III) conjugation of DOX to both ends of PCEC. After preparing PCEC (PCL_17_-PEG_34_-PCL_17_, *M*_n_ ≈ 5.3 kDa), the hydrazide-functionalized PCEC was prepared, as follows. Firstly, PCEC (1.05 g, 0.2 mmol) and TEA (83.4 μL, 0.6 mmol) were dissolved in flask with appropriate THF (10 mL). After incubation in the ice-water bath, NPC (96.7 mg, 0.48 mmol) dissolved in THF (5 mL) was dropwise added into the flask and the reaction lasted for 8 h. The resultant salts were removed by filtration and then the NPC-activated PCEC was precipitated from cold ether and dried in vacuum. Subsequently, the NPC-activated PCEC (about 1.13 g, 0.2 mmol) was dissolved in DMF and reacted with Boc-NHNH_2_ (66.1 mg, 0.5 mmol) for 12 h. The resultant mixture was treated by TFA (2.5 mL) for 4 h at room temperature to remove -COOC(CH_3_)_3_ to obtain hydrazide-functionalized PCEC. After removing DMF by dialysis in water using a dialysis membrane with a molecular weight cutoff (MWCO) of 3.5 kDa, the hydrazide-functionalized PCEC was obtained by lyophilization. Finally, hydrazide-functionalized PCEC (1.08 g, 0.2 mmol) and excess DOX (0.272 g, 0.5 mmol) were dissolved in DMSO (20 mL) and reacted for 24 h at 30 °C in the presence of acetic acid as catalyst (0.5 mL). Unreacted DOX were removed using dialysis for 24 h against DMSO. Then, DMSO was removed by dialyzing for 24 h against PBS buffer (pH 7.4). After lyophilization, DOX-*h*-PCEC was obtained at a yield of 89%. All of the reactions were performed in a dark room.

CRGDK-conjugated poly (ethylene glycol)-*b*-poly (ε-caprolactone) (CRGDK-PEG-PCL, as active targeting model) was prepared via a maleimide-thiol reaction between CRGDK and maleimide-terminated PEG-PCL. Firstly, Mal-PEG_41_-PCL_17_ was synthesized by ring opening polymerization of ε-caprolactone using Mal-PEG_41_-OH as the initiator and Sn(Oct)_2_ as the catalyst. Briefly, Mal-PEG_41_-OH (0.4 g, 0.2 mmol), ε-caprolactone (0.387 g, 3.4 mmol) and Sn(Oct)_2_ (0.091 mL) were placed in the dry flask. All of the reactants were dissolved in toluene (5 mL) and the reaction was placed in 100 °C oil bath. The reaction was carried out for 8 h under nitrogen atmosphere. The synthesized polymer was obtained by precipitation in ice-cooled diethyl ether. The resultant Mal-PEG_41_-PCL_17_ was obtained after filtration and drying at 35 °C in vacuum. Subsequently, CRGDK (57.8 mg, 0.1 mmol) and Mal-PEG_41_-PCL_17_ (0.39 g, 0.1 mmol) were directly dissolved in DMF and were then stirred for 24 h at room temperature. The product was purified by dialysis method for 24 h. CRGDK-PEG-PCL was obtained after lyophilization in a 93% yield.

The obtained DOX-*h*-PCEC and CRGDK-PEG-PCL were confirmed by using nuclear magnetic resonance (^1^H NMR) using NMR spectrometer (VARIAN INOVA 500MHz NMR spectrometer, Varian, Palo Alto, CA, USA). DOX-*h*-PCEC and CRGDK-PEG-PCL were dissolved in dimethyl sulfoxide-d_6_. The content of conjugated DOX in DOX-*h*-PCEC was quantified by UV-Vis spectrophotometer at 485 nm using WFZ-26A UV-vis spectrophotometer (Tianjin Science Instrument Plant, Tianjin, China) after hydrolyzing the hydrazone bond between DOX and PCEC under acidic conditions, according to a previous work [46]. The successful preparation of CRGDK-PEG-PCL was further confirmed by an Agilent 1100 series gel permeation chromatography (GPC) analyses (Agilent Technologies, Palo Alto, CA, USA), using Shodex GPC KF-803L column with molecular weight range 500–42,000 Da. Pure DMF was used as eluent at a flow rate of 1.0 mL/min at 30 °C.

### 2.3. Preparation and Characterization of CUR/DOX-h-PCEC@CRGDK NPs

The CUR/DOX codelivery nanomedicine CUR/DOX-*h*-PCEC@CRGDK NPs were prepared by solvent displacement method. Briefly, DOX-*h*-PCEC (9 mg), CRGDK-PEG-PCL (1 mg), and appropriate amount of CUR was completely dissolved in DMSO (1 mL). Then, the solution was added dropwise to 10 mL deionized water under magnetic stirring. The solution was placed in a dialysis bag (MWCO = 3.5 kDa) and dialyzed against water to remove DMSO and free substance at room temperature. With similar procedure, DOX-*h*-PCEC NPs, CUR/DOX-*h*-PCEC NPs, DOX-*h*-PCEC@CRGDK NPs, and CUR loaded CRGDK-PEG-PCL NPs were prepared by using DOX-*h*-PCEC, DOX-*h*-PCEC, together with CUR, DOX-*h*-PCEC, together with CRGDK-PEG-PCL, CRGDK-PEG-PCL, together with CUR, respectively. In addition, CUR/DOX coloaded PCEC NPs were also prepared with similar procedure by using PCEC together with different amount of CUR and DOX (PCEC:DOX:CUR in feed solution = 100:10:10, *w*/*w*/*w*).

The amount of DOX in the several NPs was determined by UV–vis spectrophotometer (Tianjin Science Instrument Plant, Tianjin, China) (absorption at 550 nm, minimizing the effect of CUR on UV measurement) using a standard curve method, according to a previous work [48]. The amount of CUR in a variety of NPs prepared as mentioned above was determined by HPLC (Agilent Technologies 1200 Series, Santa Clara, CA, USA) on a Kromasil-C18 column (4.6 mm × 250 mm, 5 μm) with a UV-vis detector at wavelength of 425 nm. 1 mg of lyophilized CUR encapsulated NPs was dissolved in 10 mL acetonitrile. It was analyzed by a mobile phase of acetonitrile and 3% acetic acid aqueous solution in the ratio of 70:30 (*v*/*v*) at a flow rate of 1 mL/min at 25 °C. Then, the amount of CUR can be achieved by the integral area of HPLC using a standard curve method. Drug loading content (DLC) and drug loading efficiency (DLE) were calculated from the following equations:

(1) $$\mathrm{DLC}\text{\%}=\frac{\mathrm{weight}\;\mathrm{of}\;\mathrm{loaded}\;\mathrm{drug}}{\mathrm{weight}\;\mathrm{of}\;\mathrm{drug}-\mathrm{loaded}\;\mathrm{NPs}}\text{×}100\%$$

(2) $$\mathrm{DLE}\text{\%}=\frac{\mathrm{weight}\;\mathrm{of}\;\mathrm{loaded}\;\mathrm{drug}}{\mathrm{weight}\;\mathrm{of}\;\mathrm{drug}\;\mathrm{in}\;\mathrm{feed}}\text{×}100\%$$

The size and size distribution as well as zeta potential of the NPs were measured by dynamic light scattering (DLS) (Malvern Zetasizer Nano ZS, Malvern, UK). The stability of NPs with different pH value was measured through sizes by DLS too. Transmission electron microscopy (TEM) was used to observe the morphology of NPs, using a JEM-100CX II instrument (JEOL LTD, Tokyo, Japan). The samples were prepared by adding few drops of the nanoparticle dispersion on the TEM copper grid. To examine the hemodynamic stability of the obtained CUR/DOX-*h*-PCEC@CRGDK NPs, we suspended CUR/DOX-*h*-PCEC@CRGDK NPs in PBS (pH 7.4) containing 5% bovine serum albumin (BSA) at 37 °C, and then the particle size change was monitored by DLS.

The encapsulation stability of CUR in CUR/DOX-*h*-PCEC@CRGDK NPs and CUR loaded PCEC NPs was comparatively studied by an ultrafiltration centrifugal tube. The CUR encapsulated NPs were diluted in PBS pH 7.4 and 5.0 containing 10% methanol as solubilizer, and then stored for 0 h, 24 h, and 48 h. After being filtered using a Millipore Amicon Ultra-0.5 centrifugal filter (MWCO ≈ 10 kDa, Millipore Corp., Bedford, MA, USA), the UV absorption curves of the filtrate that were collected at a different time were measured by UV-vis spectrophotometer. The changes in size of CUR/DOX-*h*-PCEC@CRGDK NPs due to acid-triggered hydrolysis of hydrazone bonds were followed by DLS. The NPs solutions in PBS pH 7.4 and 5.0 were prepared, and then the changes in NPs size at 37 °C were monitored in time by DLS.

### 2.4. In Vitro Drug Release

The release of drugs from the above NPs was studied using the dialysis method. 2 mL NPs solution was added into a dialysis bag (MWCO = 3.5 kDa) and then placed in a glass tube containing 25 mL of PBS solution. Either DOX or CUR release from NPs (dual drug loaded formulation) was investigated in PBS with different pH (pH = 6.5 and 7.4) or acetate buffer (pH = 5.0) containing 1% *v*/*v* of Tween 80. The in vitro release was placed in incubator shaker under shaking (70 rpm) at 37 °C. At the appropriate time intervals, 5 mL of the release medium was removed and replaced with equivalent fresh medium. The amount of DOX (Retention time ≈ 5.6 min) and CUR (Retention time ≈ 11.2 min) was determined by HPLC as a gradient elution method. The cumulative drug release percentage was calculated using the following equation:

(3) $$C_{r}\left(\;\%\right)=\frac{V_{t}\sum_{1}^{n-1}C_{i}{+V}_{0}C_{n}}{m_{\mathrm{drug}}}\text{×}100\%$$

where *C*_r_ is the cumulative release amount; *m*_drug_ was the amount of drug in the formations; *V*_0_ represented the whole volume of the release medium (*V*_0_ = 25 mL); *V*_t_ was the volume of the replaced medium (*V*_t_ = 5.0 mL); and, *C*_n_ was the concentration of drug in the sample.

### 2.5. In Vitro Cell Uptake

The HUVEC cells and MCF-7/ADR cells were seeded into a 12-well plate at a density of 1.0 × 10^5^ cells per well and were incubated at 37 °C for 24 h. After that, the cells were incubated with 50 nM Lyso-Tracker Green and DAPI for 30 min and 60 min, respectively (MCF-7/ADR cells were only incubated with DAPI). After washing three times with PBS, the cells were incubated with fresh medium containing of several formulations: (A) free DOX; (B) free CUR; (C) physical mixture of DOX and CUR; (D) DOX-*h*-PCEC NPs; and, (E) CUR/DOX-*h*-PCEC@CRGDK NPs. The concentration of different NPs was at an equivalent DOX dosage of 10 μg/mL. After incubation for 4 h at 37 °C, the cells were washed with PBS. The cellular uptake was visualized by confocal laser scanning microscope (Leica Microsystems, Heidelberg, Germany). The gray value was used to achieve semi-quantitative analyses by software Image-Pro Plus 6.0 (Media Cybernetics, Inc., Silver Spring, MA, USA).

### 2.6. In Vitro Cytotoxicity Study

MTT assay was performed to compare the cytotoxicity of free DOX, physical mixture of DOX and CUR, and CUR/DOX-*h*-PCEC@CRGDK NPs against MCF-7/ADR cells. MCF-7/ADR cells were seeded into the 96-well plates at 8000 cells per well, cultured in 100 μL complete dulbecco’s modified eagle medium (DMEM) with the addition of DOX (1 μg/mL), and incubated at 37 °C with 5% CO_2_. The supernatants were discarded, and the cells were washed twice with PBS (pH 7.4) after 24 h. Free DOX, physical mixture of DOX and CUR, and CUR/DOX-*h*-PCEC@CRGDK NPs were put in the wells for 24 h at a DOX concentration of 0.125, 1.25, 2.5, 5, 10, 20, 40 μg/mL, respectively. The culture medium without DOX (PBS) was put in the wells for control groups. Then, 20 μL MTT dye (5 mg/mL) was added. Then, the cells were incubated for 4 h at 37 °C and the medium was replaced by 150 μL DMSO to dissolve the resulting formazan crystals. The absorption was recorded at 570 nm using a microplate reader (Thermo Scientific Varioskan Flash, Waltham, MA, USA). The experiments were conducted in triplicate and the results were presented as the average ± standard deviation.

Cell viability rate (%) was calculated as the following Equation (4):

(4) $$\mathrm{Cell}\;\mathrm{Viability}\left(\%\right)=\frac{I_{0}{-I}_{1}}{I_{0}}\text{×}100\%$$

*I*_0_ was the absorbance of the cells incubated with the culture medium. *I*_1_ was the absorbance of the cells incubated with different formulations. The median inhibitory concentration (IC_50_) was calculated using the software GraphPad Prism 5 (GraphPad software Inc., San Diego, CA, USA).

## 3. Results and Discussion

### 3.1. Preparation and Characterization of DOX-h-PCEC and CRGDK-PEG-PCL

**Scheme 2:**
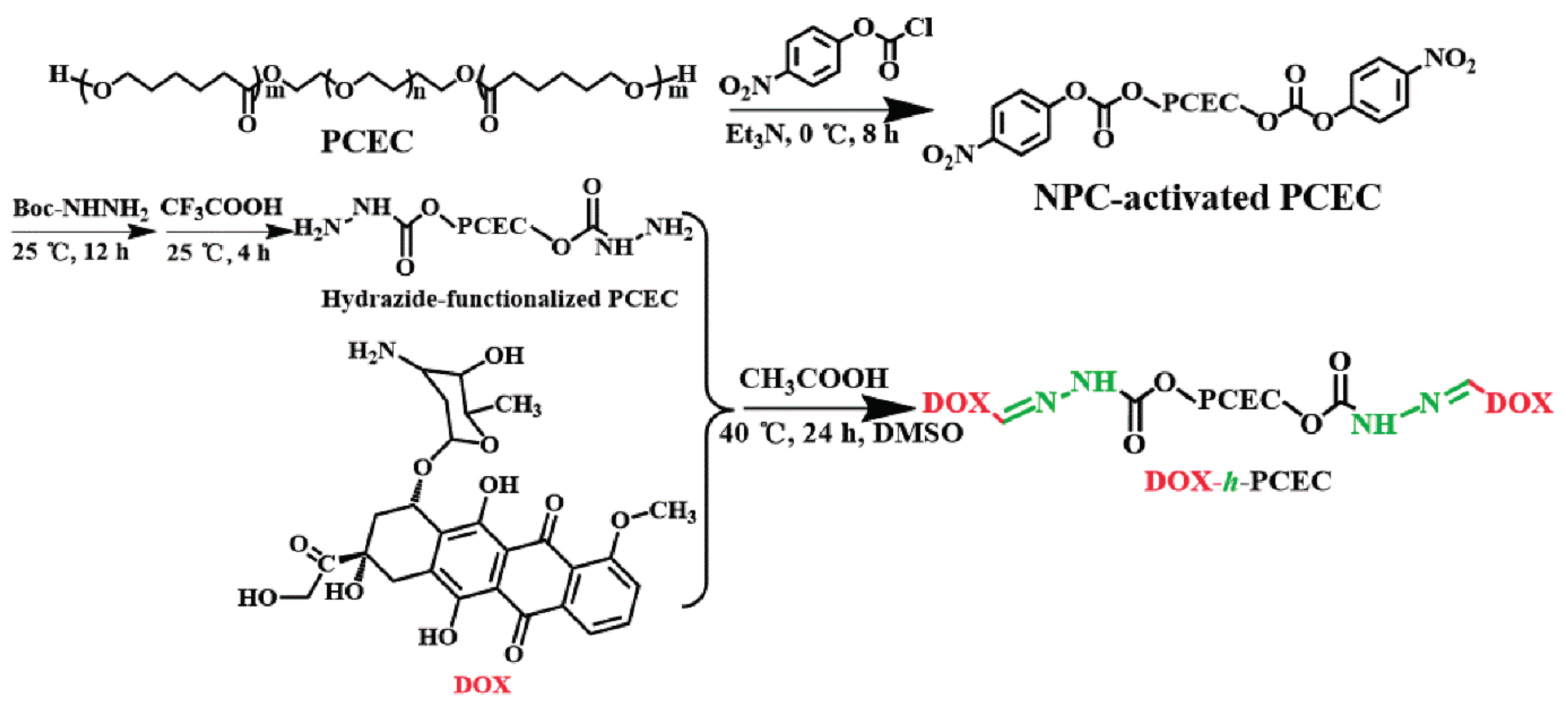
Synthetic route of DOX-*h*-PCEC.

**Figure 1:**
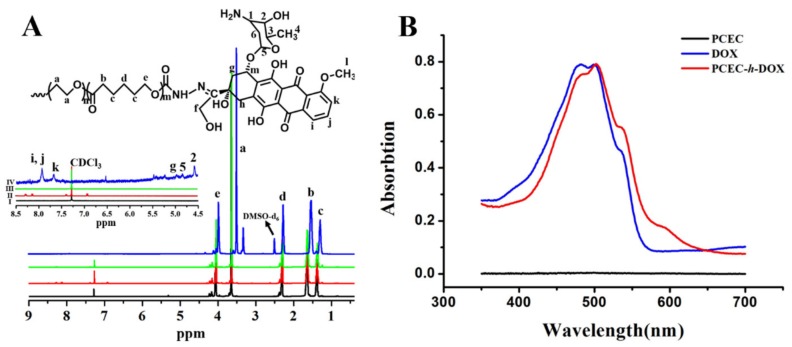
Characterization of DOX-h-PCEC. (**A**) ^1^HNMR spectra of PCEC in CDCl_3_ (I), NPC-activated PCEC in CDCl_3_ (II), Hydrazide-functionalized PCEC in CDCl_3_ (III) and DOX-*h*-PCEC in DMSO-d_6_ (IV); (**B**) UV spectra of PCEC (0.3 mg/mL), DOX (50 μg/mL), and DOX-*h*-PCEC (0.3 mg/mL) in DMSO.

The lyso/endosomal pH-sensitive macromolecular prodrug NPs derived from drug molecules covalently conjugated to the polymer chains via a cleavable hydrazone can exhibit a variety of superiorities over physical drug entrapment, such as improving total drug loading, minimizing drug leakage during circulation, and intracellular rapid drug release, which have received tremendous attention for cancer chemotherapy [46,49,50]. The block copolymers composed of PEG and PCL as building block, in our opinion, should be one of the best choices for the construction of prodrug and codelivery system, due to their adjustable amphiphilicity for self-assembly into NPs, excellent biocompatibility and biodegradability, as well as stealth-shielding property of PEG [46,51,52]. Triblock copolymers PCL-PEG-PCL, i.e., PCEC, were selected in this study, offering one more terminal group for conjugation of DOX, and thus increasing total drug payload. For preparing hydrazone-linked DOX prodrug, the terminal hydroxyl groups of PCEC should be converted to hydrazide moieties, and reacted with the ketonic groups of DOX to produce DOX-*h*-PCEC. In some previous studies [46,53,54], hydrazine monohydrate was often used to activate the end groups of polymer precursor. However, we found that the partial hydrolysis of PCEC will occur when hydrazine monohydrate was used to activate PCEC. Thus, the hydrazide-functionalized PCEC was prepared by the combinational activation of NPC and tert-butyl carbazate (Boc-NHNH_2_) and subsequent deprotection by TFA (CF_3_COOH), as shown in Scheme 2. Finally, DOX-*h*-PCEC were obtained after the reaction between the hydrazide groups of PCEC and the ketonic groups of DOX. The obtained NPC-activated PCEC and hydrazide-functionalized PCEC as well as DOX-*h*-PCEC were characterized by ^1^HNMR, as shown in Figure 1A. When compared with the ^1^HNMR spectrum of original PCEC, no significant changes in the characteristic peaks corresponding to the PEG at about 3.6 ppm and PCL moieties at about 1.0–2.2 ppm in the ^1^HNMR spectra of functionalized PCEC were observed. Meanwhile, the characteristic NPC peaks at about 8.1–8.3 in the spectrum of NPC-activated PCEC and the characteristic phenyl peaks of DOX at about 7.5–8.0 in the spectrum of DOX-*h*-PCEC can be observed. These results indicated that the terminal functionalized PCEC and DOX-*h*-PCEC were successfully obtained, simultaneously minimizing the PCEC hydrolysis. The peak intensities of ethylene protons (at about 3.6 ppm) of the PEG and the phenyl hydrogen protons of DOX (at about 8.0 ppm) can be used to evaluate the conjugated DOX content in DOX-*h*-PCEC. The DOX content in DOX-*h*-PCEC is about 15.2 wt % (the molar conjugation efficacy of DOX to PCEC chain is about 90%). In addition, the appearance of a strong DOX absorption peak at about 490 nm in the UV spectrum of DOX-*h*-PCEC indicated the successful conjugation of DOX to PCEC (Figure 1B). The UV peak intensity also indicated that the conjugation efficacy of DOX on PCEC was about 90%, which was well consistent with the result of ^1^HNMR data.

**Figure 2:**
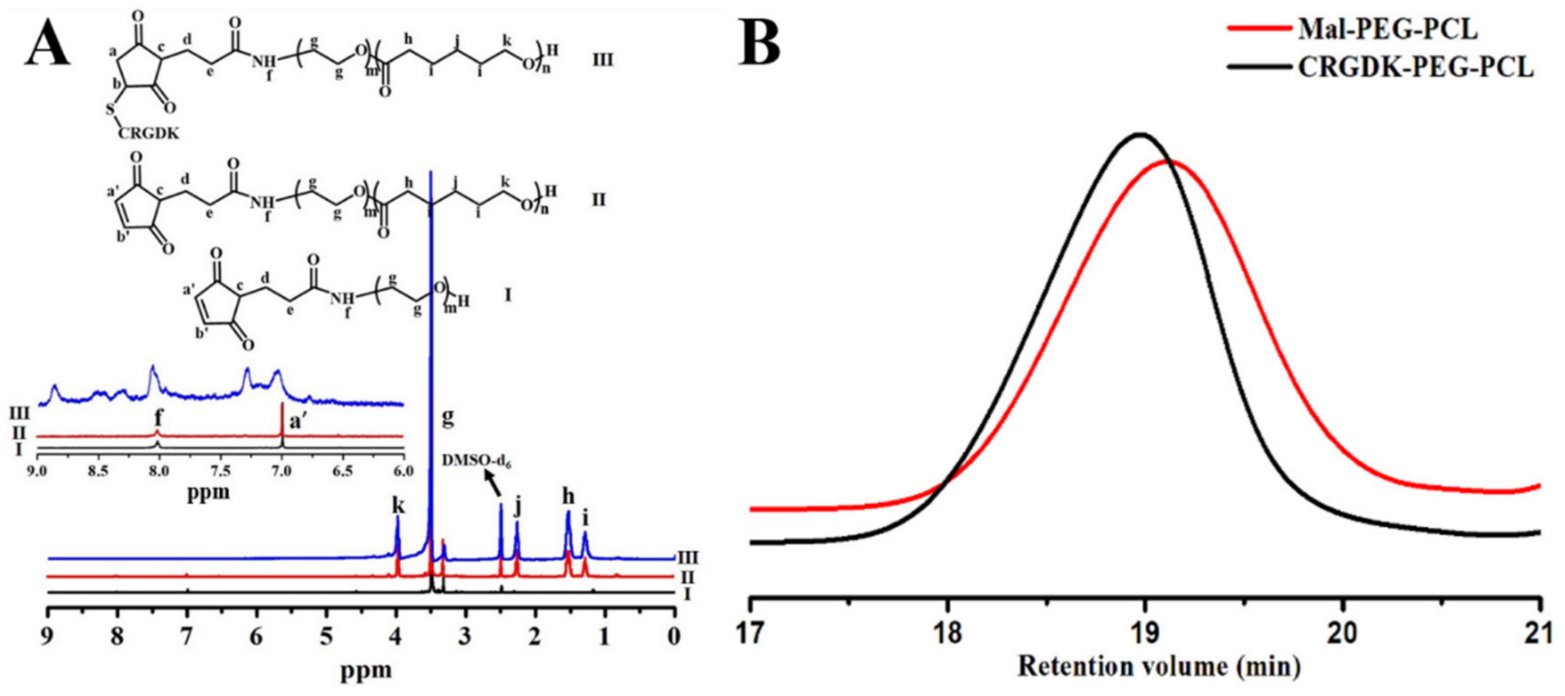
Characterization of CRGDK-PEG-PCL. (**A**) ^1^HNMR spectra of Mal-PEG (I), Mal-PEG-PCL (II) and CRGDK-PEG-PCL (III) in DMSO-d_6_; (**B**) gel permeation chromatography (GPC) curves of Mal-PEG-PCL and CRGDK-PEG-PCL in DMF.

CRGDK-PEG-PCL was prepared by an efficient maleimide-thiol reaction between thiol group of CRGDK and maleimide group of Mal-PEG-PCL at room temperature. To confirm the coupling between CRGDK and Mal-PEG-PCL, ^1^HNMR was first used to analyze the product. As shown in Figure 2A, in addition of the characteristic peaks corresponding to the PEG at about 3.6 ppm and PCL moieties at about 1.0–2.2 ppm, the ^1^HNMR spectrum of CRGDK-PEG-PCL shows the characteristic peaks corresponding to the CRGDK segment in the range of 6.5–9.0 ppm, indicating the coupling between PEG-PCL polymer chain and the CRGDK moiety. To further confirm the conjugation reaction, GPC was used to analyze the product. Both Mal-PEG-PCL and CRGDK-PEG-PCL possess a monomodal molecular weight distribution and low polydispersity. When compared to Mal-PEG-PCL, the curve of the conjugated product shifted to a lower retention time, indicating an increase in molecular weight, and thus demonstrating the successful conjugation of CRGDK to Mal-PEG-PCL.

### 3.2. Preparation and Characterization of CUR/DOX-h-PCEC@CRGDK NPs

The coassembly of two or more components has demonstrated to be a simple and efficient method to construct well-defined nanostructures with diverse structures, morphologies, and functions towards applications in drug delivery [26,55,56,57,58]. In addition, this strategy was also considered to be effective approach to ratiometrically control multidrug loading [19,26,56,57]. The amphiphilic DOX-*h*-PCEC and CRGDK-PEG-PCL cannot only self-assemble into NPs, but also coassemble into nanoformulations together with or without additional hydrophobic drugs. Apparently, the integration of DOX-*h*-PCEC as drug vector model, CRGDK-PEG-PCL as active targeting model and appropriate amount of hydrophobic CUR as MDR modulator model, via coassembly approach, will generate CUR/DOX-coloaded, CRGDK-targeted, and MDR-inhibited combinational drug delivery. A series of DOX, CUR, and CUR/DOX loaded NPs were successfully prepared by ratiometric coassembly of a different model. Their physicochemical properties were characterized in detail, as shown in Table 1. In our experimental condition, the results showed that all CUR/DOX-*h*-PCEC@CRGDK NPs showed remarkably higher encapsulation efficiencies toward CUR, especially at a relatively low CUR dose in feed solution. Even in the case of high CUR dose in feed solution (about 20%), the DLE of CUR/DOX-*h*-PCEC@CRGDK NPs is about 71.4%, which is much higher than that of CUR loaded CRGDK-PEG-PCL. These phenomena should be ascribed to the combination of hydrophobic interaction between PCL and CUR with strong intermolecular π−π stacking action of DOX benzene in DOX-*h*-PCEC and CUR [34,59,60]. Moreover, as shown in the Table 1, the DLC of CUR in CUR/DOX-*h*-PCEC@CRGDK NPs can be ratiometrically controlled in a dose-depended manner. In addition, the results indicated that all of the DOX, CUR, and CUR/DOX loaded NPs by modular coassembly had low PDI of 0.11−0.23 and particle sizes ranging from 95 ± 6 to 135 ± 11 nm, depending on the NPs composition and CUR loading levels.

**Figure 3:**
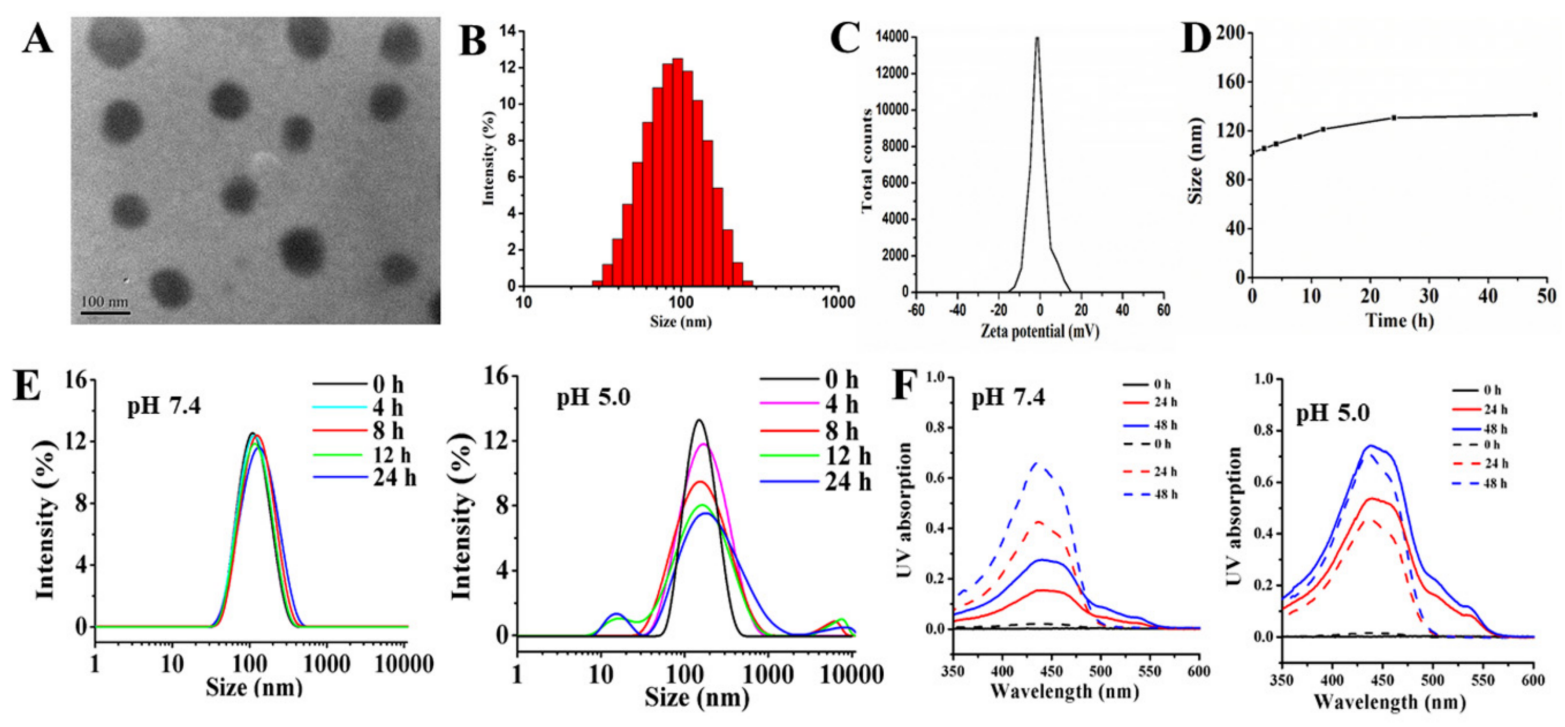
Characterizations of CUR/DOX-*h*-PCEC@CRGDK NPs. (**A**) TEM image; (**B**) size histogram; (**C**) Zeta potential at pH 7.4; (**D**) Hemodynamic stability in pH 7.4 PBS containing 5% BSA at 37 °C; (**E**) Size changes of NPs incubated in PBS of pH 7.4 and pH 5.0; (**F**) CUR leakage from CUR/DOX-*h*-PCEC@CRGDK NPs (solid line) and CUR loaded CRGDK-PEG-PCL NPs (dashed line) incubated in PBS of pH 7.4 and pH 5.0 containing 10% methanol as solubilizer; The leaked CUR samples were diluted 10 times by same incubation solution.

Typically, the CUR/DOX-*h*-PCEC@CRGDK NPs prepared by ratiometric coassembly of DOX-*h*-PCEC, CRGDK-PEG-PCL and CUR at a predetermined dose ratio (90:10:10, *w*/*w*/*w*), unless specifically indicated, were further characterized for their properties and performances. As shown in Figure 3A,B, CUR/DOX-*h*-PCEC@CRGDK NPs exhibited spherical morphology with a uniform size distribution. Figure 3C indicated that the CUR/DOX-*h*-PCEC@CRGDK NPs displayed a neutral zeta potential of 0.41 ± 0.12 mV, which should be conductive to avoid NPs being cleared by kidneys infiltration. The variation of particle size in BSA-containing PBS was often used to reflect the hemodynamic stability of nanocarriers. The size change as a function of incubation time for CUR/DOX-*h*-PCEC@CRGDK NPs in PBS of pH 7.4 with 5% BSA at 37 °C is shown in Figure 3D. No significant variation was observed during the entire time of observation. It indicated no occurrence of particle aggregation, and thus high colloidal stability. The good stability can be ascribed to that the steric stabilization of PEG shell. Figure 3E further indicated the physical stability of CUR/DOX-*h*-PCEC@CRGDK NPs at pH 7.4, as no any significant variation of particle size was observed over 24 h at pH 7.4. However, under otherwise the same conditions, the considerable expansion of NPs at pH 5.0 was observed. The hydrazone bonds between PCEC and DOX could be specifically hydrolyzed in the acidic environment, which would lead to the disassembly of NPs and the acceleration of the drug release. In addition, the conjugation of drug molecules to the PCL chain has been proven to be able to disturb the PCL chain regularity, hereby decreasing the crystallization of the PCL segments and enhancing the acid-triggered disassembly of NPs with PCL as the core-forming block [46,47]. The CUR/DOX-*h*-PCEC@CRGDK NPs were incubated in different pH media for a predetermined time, followed by filtration through an Amicon ultrafilter to collect the released CUR. The corresponding CUR loaded CRGDK-PEG-PCL NPs were tested in parallel as a control group. It was found that a significantly lower amount of CUR was leaked out of the CUR/DOX-*h*-PCEC@CRGDK NPs as compared to the CUR loaded CRGDK-PEG-PCL NPs at pH 7.4 (Figure 3F). Their higher capability to effectively retain encapsulated CUR indicated the greater stabilization due to the formation of stronger interaction, i.e., intermolecular π−π stacking action between DOX and CUR in inner hydrophobic core of CUR/DOX-*h*-PCEC@CRGDK NPs. However, under acidic condition (pH 5.0), higher UV intensity assigned to the leakage of CUR (440 nm), and DOX (>500 nm) were observed, further demonstrating the pH-sensitiveness of CUR/DOX-*h*-PCEC@CRGDK NPs. The results also confirmed that the acid-enhanced hydrolysis of the hydrazone linkage could cause rapid and simultaneous release of DOX and CUR. Apparently, the simultaneous corelease of DOX and CUR in response to the tumor intracellular acidic microenvironment is highly desirable for the effective treatment of cancer.

### 3.3. In Vitro Drug Release

**Figure 4:**
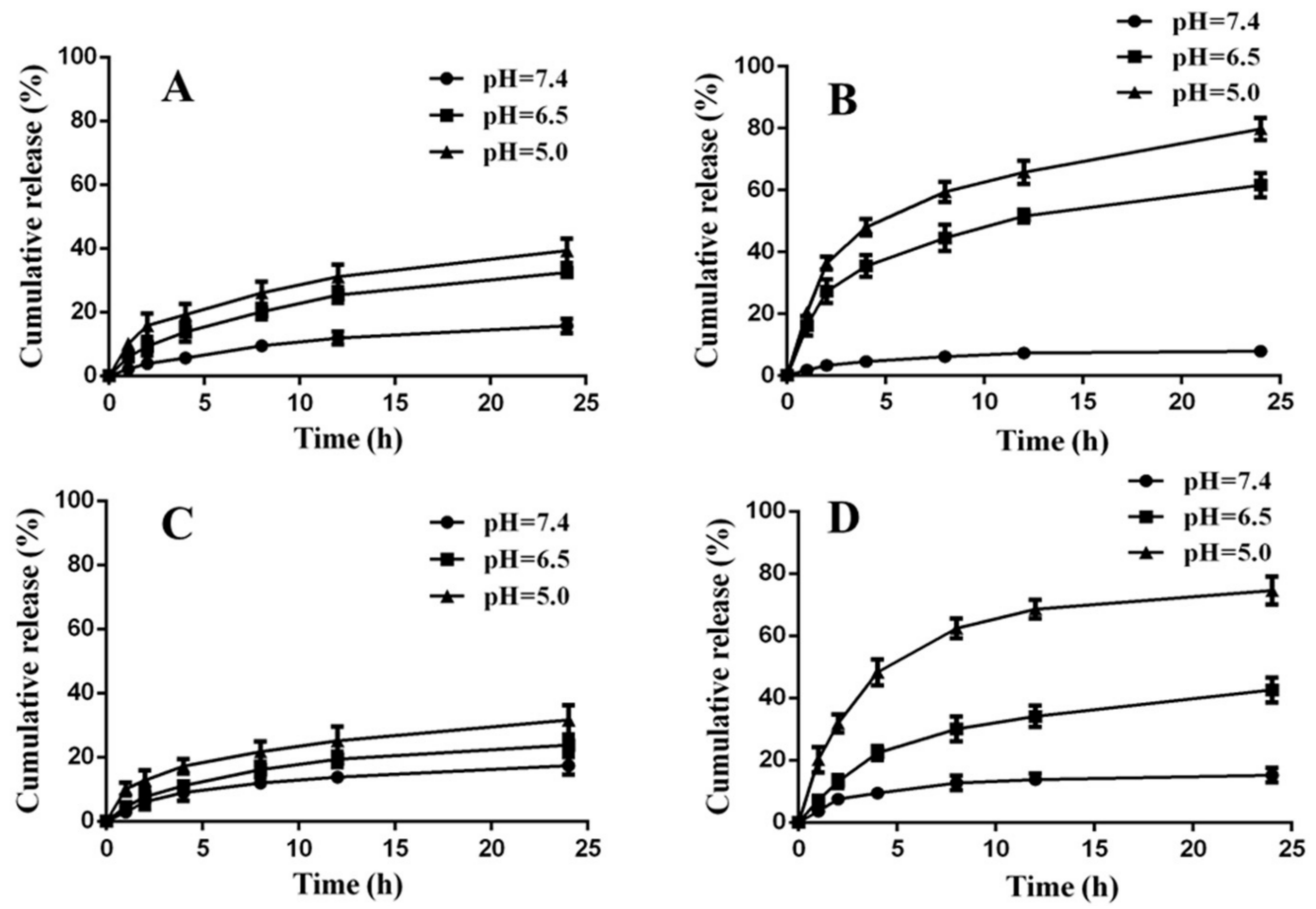
In vitro drug release profiles. (**A**) DOX release from CUR/DOX coloaded PCEC NPs; (**B**) DOX release from CUR/DOX-*h*-PCEC@CRGDK NPs; (**C**) CUR release from CUR/DOX coloaded PCEC NPs; (**D**) CUR release from CUR/DOX-*h*-PCEC@CRGDK NPs;.

The in vitro release profiles of DOX and CUR from CUR/DOX-*h*-PCEC@CRGDK NPs and CUR/DOX coloaded PCEC NPs as control were comparatively studied at 37 °C at pH 7.4 (the extracellular pH of normal tissue), 6.5 (the extracellular environment of tumor tissues), and 5.0 (lyso/endosomal environment of tumor cells) using a dialysis method, as shown in Figure 4. Both the release rate of DOX and CUR from CUR/DOX coloaded PCEC NPs at pH 7.4 was relatively low, parallel to each other throughout the study (Figure 4A,C). Only approximately 15% of drug was released from PCEC NPs within 24 h. With the decrease of the pH values, no marked change for CUR release from PCEC NPs (Figure 4C). However, when compared with the CUR release from PCEC NPs, the release of DOX from PCEC NPs was slightly accelerated at pH 6.5 and pH 5.0, which may be due to the improved solubility of DOX because of DOX protonation in acidic media. The effects of pH values on the in vitro release behaviors of DOX from CUR/DOX-*h*-PCEC@CRGDK NPs are shown in Figure 4B. A significantly pH-dependent release profile was observed. The release of conjugated DOX from CUR/DOX-*h*-PCEC@CRGDK NPs was very low, at pH 7.4. Only approximately 6% of DOX was released at 24 h, which was much lower than DOX release from PCEC NPs (about 15%). These results can be due to the high stability of hydrazone bonds between DOX and polymers under physiological conditions (pH 7.4). However, the release rate of DOX was significantly increased at acidic pH values. The DOX was released about 58.6% at pH 6.5 and 78.2% at pH 5.0 within 24 h, respectively. The significant increase in DOX release can be attributed to the combination of acid-induced hydrolysis of hydrazone bond, accelerated disassembly of NPs, and enhanced solubility of DOX in acidic media. Similarly, a pH-controlled release profile of CUR from CUR/DOX-*h*-PCEC@CRGDK NPs was observed (Figure 4D). The CUR release rate was significantly faster at pH 6.5 and 5.0. About 70% of the CUR was released from CUR/DOX-*h*-PCEC@CRGDK NPs within 24 h. The significant increase in CUR release under acidic conditions can be ascribed to that the pH-induced hydrolysis of hydrazone bond can cause the swelling and disassembly of CUR/DOX-*h*-PCEC@CRGDK NPs, as demonstrated in Figure 3E,F, facilitating the diffusion and release of CUR. These results further indicated that the simultaneous corelease of DOX and CUR by acid-triggered degradation of prodrug and subsequent disassembly of NPs occurred, which will be desirable to maximize the synergistic effect of DOX and CUR within the tumor cells. In sum, CUR/DOX-*h*-PCEC@CRGDK NPs presented pH-dependent DOX and CUR corelease behaviors, which should be beneficial for tumor treatment. Most of DOX and CUR will remain in NPs for a considerable length of time at normal physiological conditions (pH 7.4). However, both the loaded DOX and CUR will be rapidly and simultaneously released due to the acidic environment in lyso/endocytic compartments (pH 5.0−6.5) after uptake by tumor cells.

### 3.4. In Vitro Cell Uptake

**Figure 5:**
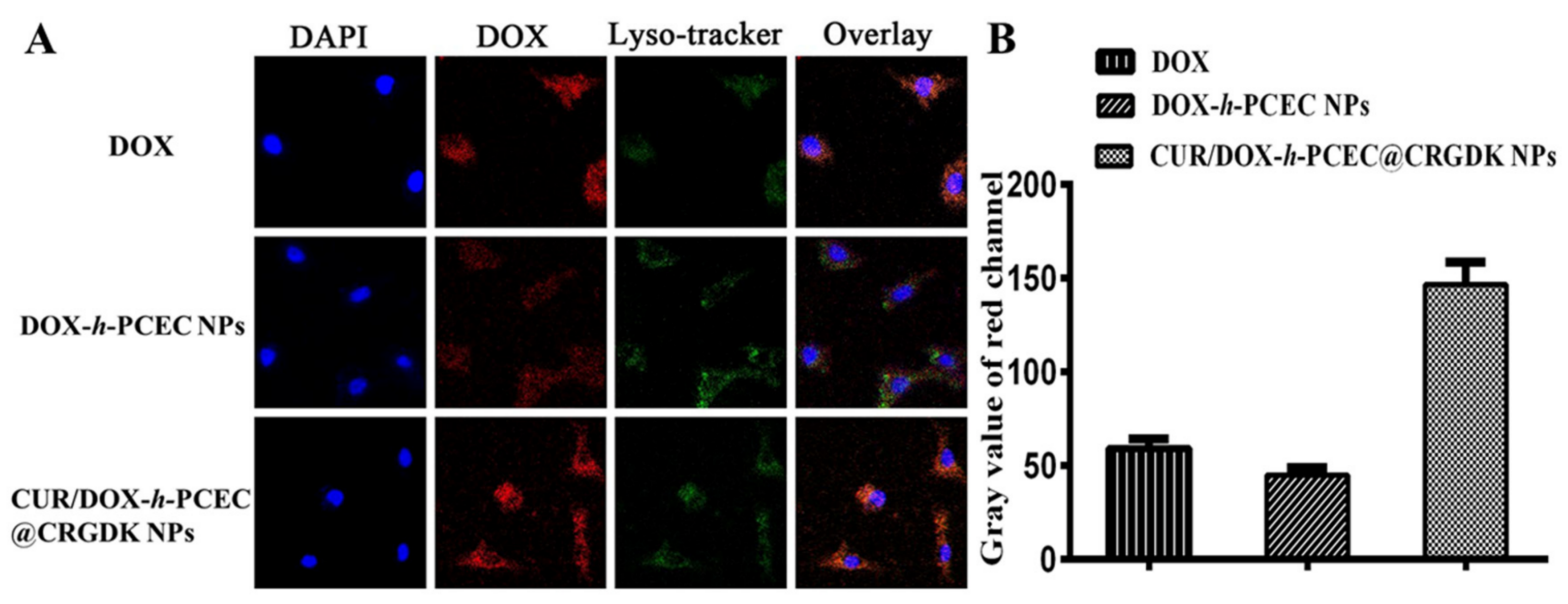
Selective cellular uptake. (**A**) Representative fluorescence microscopy images of Neuropilin-1 overexpressed human umbilical vein endothelial cells (HUVEC) cells incubated with free DOX, DOX-*h*-PCEC NPs, and CUR/DOX-*h*-PCEC@CRGDK NPs at equivalent DOX concentration of 10 μg/mL for 4 h; (**B**) Average gray value as a criterion to compare the red fluorescent intensity in different images.

Efficient and selective cellular uptake of therapeutic drugs plays a key role in improving chemotherapy efficacy and decreasing side effects. Neuropilin-1 is overexpressed in many human cancer cells, providing a useful targeting site for tumor-specific drug delivery [41,42,43,44,45]. In this study, the cellular uptake of free DOX, DOX-*h*-PCEC NPs, and CUR/DOX-*h*-PCEC@CRGDK NPs was monitored by confocal laser scanning microscope in Neuropilin-1 overexpressed HUVEC cells. DAPI was used to label nucleus (blue) and Lyso-Tracker was applied to label lysosomes (green). As shown in Figure 5A, the red signals, due to the intrinsic fluorescence of DOX, were clearly observed in the cytoplasm and nuclei of the treated HUVEC cells, indicating the rapid passive diffusion of free DOX and effective endocytosis of DOX-loaded NPs. Further inspection of Figure 5A shows that, for the cells after 4 h of incubation with free DOX, the relative weak fluorescence was observed in the cytoplasm of cells, indicating that the DOX molecules successfully entered the cells, but mainly accumulated in the cytoplasm. By contrast, when the cells were incubated with DOX-loaded NPs, the fluorescence mainly appeared in the proximity of cellular nuclei. When compared with fluorescence intensity in the cells that were treated with DOX-*h*-PCEC NPs, the detected fluorescence in the cells treated with CUR/DOX-*h*-PCEC@CRGDK NPs was much higher in the nuclei. Apparently, significantly enhanced cellular uptake and considerably increased nucleus localization were confirmed in HUVEC cells that were incubated with CUR/DOX-*h*-PCEC@CRGDK NPs. Moreover, as a measurement of fluorescence intensity of DOX, the gray value for images, as a gross approximation, was determined using the image analysis software (Figure 5B). The results of measuring fluorescence intensity based on average gray value also demonstrated that both the cell uptake and nucleus localization of CUR/DOX-*h*-PCEC@CRGDK NPs were much higher than that of free DOX and DOX-*h*-PCEC NPs without CRGDK. These results definitely revealed that CRGDK surface decorated CUR/DOX-*h*-PCEC@CRGDK NPs possess significantly improved binding activity with Neuropilin-1 overexpressing cancer cells, and thus can exploit the efficient receptor-mediated endocytosis to achieve cell-specific drug delivery.

### 3.5. Intracellular Synergistic Corelease and Cytotoxicity

**Figure 6:**
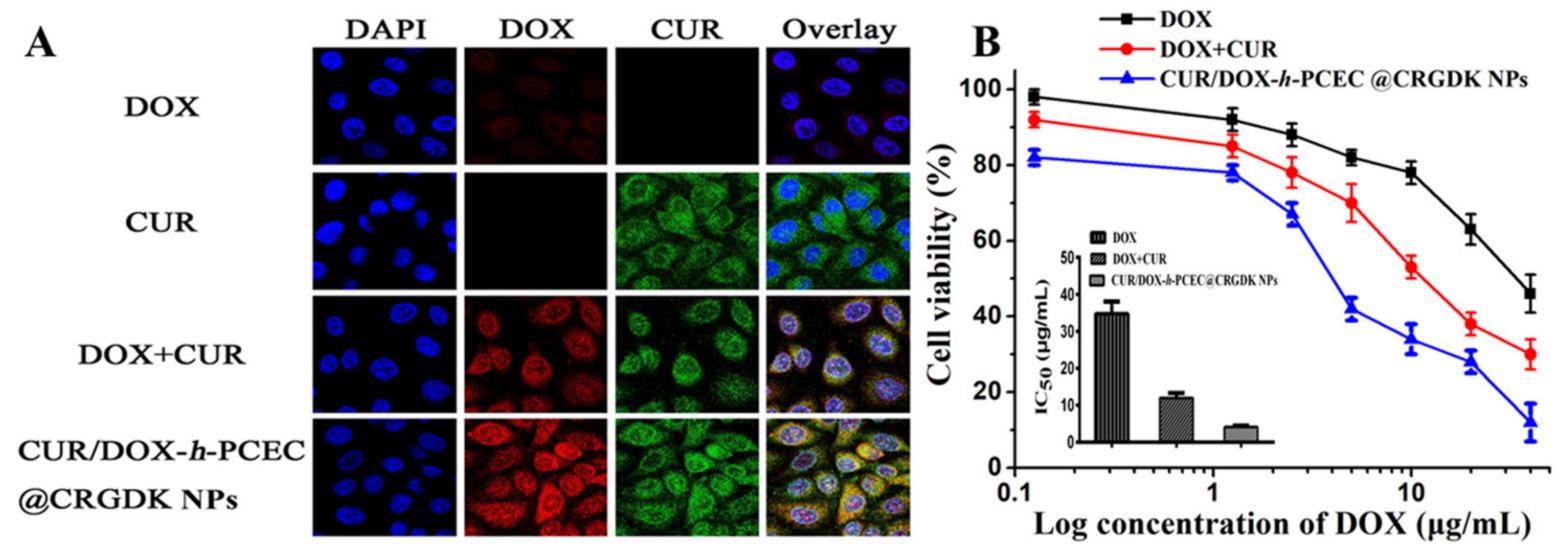
Intracellular synergistic corelease and cytotoxicity. (**A**) Representative fluorescence microscopy images of Adriamycin resistant MCF-7/ADR cells with free DOX, free CUR, physical mixture of DOX and CUR (1.0:1.0, *w*/*w*), and CUR/DOX-*h*-PCEC@CRGDK NPs at equivalent DOX concentration of 10 μg/mL for 4 h; (**B**) Cytotoxicity of free DOX, physical mixture of DOX and CUR (1.0:1.0, *w*/*w*), and CUR/DOX-*h*-PCEC@CRGDK NPs to MCF-7/ADR cells.

As is well known, the MDR cells possess drug efflux abilities by overexpressing P-gp, presenting a critical challenge to effective cancer therapy [5,8]. In the present study, to verify whether CUR/DOX-*h*-PCEC@CRGDK NPs enabled cells to overcome MDR by synergistic corelease of DOX and CUR to inhibit P-gp overexpression, we applied the intrinsic fluorescence of DOX and CUR to carry out a comparative analysis of intracellular drug localization in the drug-resistant MCF-7/ADR cells treated with free DOX, free CUR, DOX+CUR (1.0:1.0, *w*/*w*), and CUR/DOX-*h*-PCEC@CRGDK NPs. As shown in Figure 6A, no obvious intracellular accumulation of DOX within the MCF-7/ADR cells treated with free DOX was observed, presumably due to a powerful MDR effect. It can be seen that the DOX + CUR group exhibited higher intracellular DOX accumulation than free DOX group, confirming that CUR can facilitate DOX uptake into MCF-7/ADR cells. More importantly, the results in Figure 6A clearly showed that, in comparison with free DOX and DOX**+**CUR group, significantly stronger DOX and CUR fluorescence appeared within the cells treated by CUR/DOX-*h*-PCEC@CRGDK NPs. Notably, CUR/DOX-*h*-PCEC@CRGDK NPs can significantly increase the cellular uptake of both DOX and CUR in MCF-7/ADR cells, which can be attributed to the effective internalization because of the active targeting effect of CRGDK and efficient intracellular corelease due to the acid-triggered cleavage of hydrazone. In addition, these results not only suggested that the CUR/DOX-*h*-PCEC@CRGDK NPs can synergistically and simultaneously corelease DOX and CUR, but also confirmed that the nanoparticulate codelivery of DOX and CUR can more effectively inhibit the overexpression of P-gp and thus reverse the MDR, finally increasing the intracellular accumulation and retention of DOX.

To demonstrate the in vitro anticancer activity of CUR/DOX-*h*-PCEC@CRGDK NPs, we tested the cell cytotoxicity of free DOX, DOX+CUR (1.0:1.0, *w*/*w*), and CUR/DOX-*h*-PCEC@CRGDK NPs against MCF-7/ADR cells. All of the formulations gradually increased their cell cytotoxicity with the increase of concentrations. However, MCF-7/ADR cells showed the lowest sensitivity to the treatment with free DOX. The half-maximal inhibitory concentration (IC_50_) value of free DOX was measured to be about 34.7 μg/mL. The relatively high IC_50_ should be due to that MCF-7/ADR cells were highly resistant to DOX. However, DOX+CUR group had a much higher cytotoxicity to MCF-7/ADR cells (IC_50_ = 12.1 ± 0.7 μg/mL) than free DOX, due to the chmosensitization effect on MDR reversal of CUR, as mentioned above. In particular, the CUR/DOX-*h*-PCEC@CRGDK NPs presented remarkably high cytotoxicity against MCF-7/ADR cells, as their IC_50_ value was considerably decreased to 4.1 ± 0.2 μg/mL.

The CUR/DOX-*h*-PCEC@CRGDK NPs effectively corelease the encapsulated chemotherapeutic drug and chemosensitizer, thus achieving an enhanced cytotoxicity against MCF-7/ADR cells. These results further confirmed the excellent capability of CUR/DOX-*h*-PCEC@CRGDK NPs for efficient tumor targeting delivery and intracellular synergistic corelease to modulate the drug resistance.

## 4. Conclusions

In this work, we applied a modular coassembly of acid-cleavable PEGylated polymeric prodrug, tumor cell-specific targeting peptide, and natural chemosensitizer to construct an all-in-one multifunctional multidrug delivery system (CUR/DOX-*h*-PCEC@CRGDK NPs) for the synergistic codelivery of DOX and CUR. The CUR/DOX-*h*-PCEC@CRGDK NPs were demonstrated to be able to ratiometrically load DOX and CUR, allow for long circulation, enter the cells via CRGDK-receptor mediated tumor targeting, as well as realize tumor intracellular responsive and simultaneous corelease of DOX and CUR, thus effectively reversing the MDR through inhibiting P-gp expression. We believe that the design presented here can provide a facile and robust nanoplatform for targeted multidrug codelivery to maximize synergistic effects, thus exhibiting great potential for future applications in the combinatory cancer therapy.

## Figures and Tables

**Table 1:** Characterizations of DOX, CUR, and CUR/DOX loaded NPs by modular coassembly.

Samples	Weight Ratio in Feed Solution (DOX-*h*-PCEC:CRGDK-PEG-PCL:CUR)	Size ^a^ (nm)	PDI ^a^	DLC of CUR (%)	DLE of CUR (%)	DOX Content ^b^ (wt %)	CUR Content ^c^ (wt %)
**I ^d^**	**90:10:0**	**115 ± 3**	**0.16**	**-**	**-**	**13.7**	**0**
**II**	**90:10:1**	**114 ± 5**	**0.11**	**0.93**	**92.7**	**13.7**	**0.92**
**III**	**90:10:2**	**108 ± 3**	**0.12**	**1.82**	**91.1**	**13.7**	**1.79**
**IV**	**90:10:5**	**110 ± 5**	**0.10**	**4.45**	**88.9**	**13.7**	**4.26**
**V**	**90:10:10**	**98 ± 3**	**0.11**	**8.07**	**80.7**	**13.7**	**7.45**
**VI**	**90:10:20**	**105 ± 7**	**0.17**	**14.28**	**71.4**	**13.7**	**12.50**
**VII ^e^**	**100:0:0**	**118 ± 5**	**0.19**	**-**	**-**	**15.2**	**0**
**VIII ^f^**	**100:0:10**	**95 ± 6**	**0.15**	**8.23**	**82.3**	**15.2**	**7.61**
**IX ^g^**	**0:100:10**	**135 ± 11**	**0.23**	**3.76**	**37.6**	**0**	**3.62**

^a^ Determined by DLS; ^b^ Calculated by the ratio of the weight of conjugated DOX to the total weight of DOX-*h*-PCEC and CRGDK-PEG-PCL in feed; ^c^ Calculated by the ratio of the weight of encapsulated CUR to the weight of NPs; ^d^ Sample I is DOX-*h*-PCEC@CRGDK NPs; ^e^ Sample VII is DOX-*h*-PCEC NPs; ^f^ Sample VIII is CUR/DOX-*h*-PCEC NPs; ^g^ Sample IX is CUR loaded CRGDK-PEG-PCL NPs.
